# Stepwise Application of Urine Markers to Detect Tumor Recurrence in Patients Undergoing Surveillance for Non-Muscle-Invasive Bladder Cancer

**DOI:** 10.1155/2014/973406

**Published:** 2014-12-22

**Authors:** Tilman Todenhöfer, Jörg Hennenlotter, Michael Esser, Sarah Mohrhardt, Stefan Aufderklamm, Johannes Böttge, Steffen Rausch, Johannes Mischinger, Simone Bier, Georgios Gakis, Ursula Kuehs, Arnulf Stenzl, Christian Schwentner

**Affiliations:** ^1^Department of Urology, Eberhard-Karls University, Hoppe-Seyler Strasse 3, 72076 Tübingen, Germany; ^2^V6H 3Z6 Vancouver Prostate Centre, University of British Columbia, 2660 Oak Street, Vancouver, BC, Canada V6H 3Z6

## Abstract

*Background*. The optimal use of urine markers in the surveillance of non-muscle-invasive bladder cancer (NMIBC) remains unclear. Aim of the present study was to investigate the combined and stepwise use of the four most broadly available urine markers to detect tumor recurrence in patients undergoing surveillance of NMIBC. *Patients and Methods*. 483 patients with history of NMIBC were included. Cytology, UroVysion, fluorescence in situ hybridization (FISH), immunocytology (uCyt+), and NMP22 ELISA were performed before surveillance cystoscopy. Characteristics of single tests and combinations were assessed by contingency analysis. *Results*. 128 (26.5%) patients had evidence of tumor recurrence. Sensitivities and negative predictive values (NPVs) of the single tests ranged between 66.4–74.3 and 82.3–88.2%. Two-marker combinations showed sensitivities and NPVs of 80.5–89.8 and 89.5–91.2%. A stepwise application of the two-test combinations with highest accuracy (cytology and FISH; cytology and uCyt+; uCyt+ and FISH) showed NPVs for high-risk recurrences (G3/Cis/pT1) of 98.8, 98.8, and 99.1%, respectively. *Conclusions*. Combinations of cytology, FISH, immunocytology, and NMP22 show remarkable detection rates for recurrent NMIBC. Stepwise two-test combinations of cytology, FISH, and immunocytology have a low probability of missing a high-risk tumor. The high sensitivities may justify the use of these combinations in prospective studies assessing the use of urine markers to individualize intervals between cystoscopies during follow-up.

## 1. Introduction

A large proportion of patients with non-muscle-invasive bladder cancer (NMIBC) experiences recurrence of disease after surgical resection of the tumor [1]. As some of these recurrences are associated with tumor progression, a thorough follow-up of these patients is of utmost importance. Currently, cystoscopy and cytology form the gold standard for the surveillance of patients with NMIBC [2, 3]. However, the sensitivity of cystoscopy is discussed controversially and many patients perceive it as a relatively painful procedure, which potentially reduces patients' compliance [4].

The number of studies assessing the performance of urine markers to detect bladder cancer has increased rapidly, also in the context of recurrence detection of NMIBC [5–7]. However, the only marker, which is clearly recommended by current guidelines, is urine cytology [2, 8]. This is in contrast to the observation that cytology exhibits a low sensitivity for low-grade tumors. It can therefore only serve as an adjunct to cystoscopy and not replace it. The combined use of urine markers or a stepwise testing may increase the detection rate for tumor recurrence [9]. We therefore aimed to investigate the diagnostic performance of combined applications of the four most broadly available urine markers cytology, FISH, uCyt+, and NMP22 in a large cohort of patients undergoing surveillance for NMIBC.

## 2. Patients and Methods

### 2.1. Patients and Samples

483 patients undergoing surveillance of NMIBC were enrolled between August 2006 and October 2011 (406 men and 77 women, median age 70 years, range 31–94). The study was approved by the local ethics committee (number 400/2009A). Urine samples were obtained before cystoscopy. Cytology, FISH (UroVysion, Vysis, Downer's Grove, USA), uCyt+ (SCIMEDX Corporation, Denville, USA), and NMP22-ELISA Bladder Cancer Test (NMP22, Alere, San Diego, USA) were performed in all patients. All patients underwent cystoscopy and upper urinary tract imaging. All patients with suspicious findings in cystoscopy underwent transurethral resection (TUR-BT).

### 2.2. Urine Tests

Urine markers were performed as previously described [10]. For cytology urine was centrifuged and cytospinned and stained after Papanicolaou with subsequent microscopic assessment according to the recommendations of the Papanicolaou Society of Cytopathology [11]. Accepted characteristic features of urothelial carcinomas were taken into consideration: papillary clusters comprised of cells with eccentric nuclei, single cells with eccentric nuclei, increased nuclear-to-cytoplasmic ratio, cells with irregular nuclear borders, and cells with coarse chromatin. Categories for cytology were (1) benign; (2) atypia, favour reactive; (3) atypia, unclear if reactive or neoplastic; (4) suspicious; (5) positive for urothelial carcinoma; (6) suspicious for high grade urothelial carcinoma. “Benign” and “atypia, favour reactive” were considered as negative cytology whereas all other categories were considered as positive cytology. The UroVysion FISH assay was performed as previously described [12]. It was considered positive if one of the following criteria was met: (1) ≥4 out of 25 morphologically suspicious cells with ≥3 signals of at least two chromosomes 3, 7, and 17; (2) ≥12 nuclei with homozygous loss of 9p21 [13]. The uCyt+ test was performed as recently described and considered positive if at least one cell showed a positive signal of CEA or mucin [14, 15]. The NMP22-ELISA test was performed according to the manufacturer's protocol with a threshold of 10 IU/mL [16].

### 2.3. Statistical Analysis

First, contingency analyses were performed to determine sensitivities, specificities, and negative and positive predictive values of single urine markers to detect tumor recurrence. Furthermore, these parameters were determined for combinations of two, three, and four urine markers by contingency analysis. For analysis of urine marker combinations, two ways of considering a combination positive were analyzed: (1) marker combination positive if at least one marker had a positive test result; (2) marker combination positive if all markers were positive.

As a stepwise testing might reduce costs compared to a simultaneous testing of markers, we simulated the performance of stepwise testing for the two-marker combinations with the highest accuracy. For stepwise testing, the second marker was only determined in patients in the case the first marker was negative.

## 3. Results

A total of 128 patients (26.5%) had evidence of tumor recurrence at time of urine marker testing. The median interval between last tumor and urine marker sampling was 6 months (3–94). Tumor stages at time of recurrence were as follows: 86 patients (67.2%) had a pTa tumor, 15 patients (11.7%) a pT1 tumor, and 14 patients (10.9%) a muscle invasive tumor (≥pT2). Grading was G1 in 53 patients (42.4%), G2 in 38 patients (29.7%), and G3 in 25 patients (19.5%). 21 patients (17.9%) had carcinoma in situ. Patients' characteristics are summarized in Table 1.

**Figure 1:**
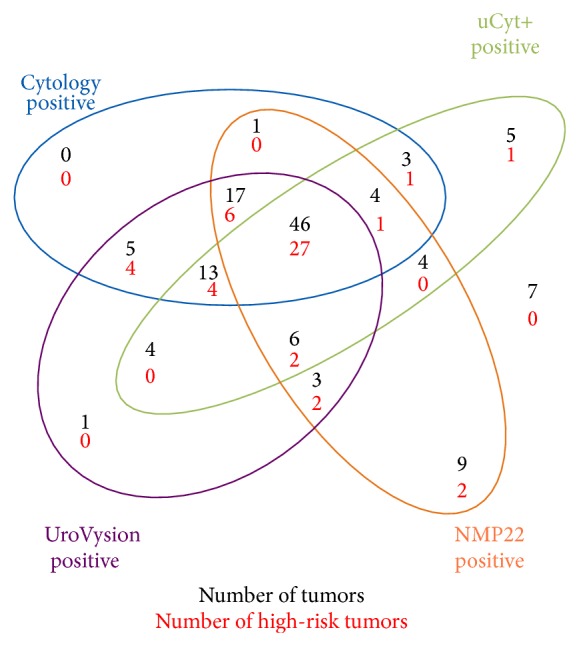
*Urine marker results in patients with tumors and high-risk tumor (≥pT1 or G3 or Cis).* Coloured circles contain number of patients with positive urine markers.

Urine marker results of patients with tumor and high-risk tumor (≥pT1 or G3 or Cis) are shown in Figure 1.

### 3.1. Single Tests

Sensitivities, specificities, PPVs, NPVs, and accuracies for the four tests are summarized in Table 2. In conclusion, FISH had best sensitivity (74.3%) whereas cytology had best specificity (75.5%). Accuracy was best for cytology (73.3%).

### 3.2. Two-Test Combinations

Performances of two-test combinations with different criteria for a positive combination are shown in Table 3. When considering a combination positive if at least one marker was positive, increased sensitivities but decreased specificities were observed compared to the single tests. The two-test combination with best sensitivity was uCyt+ and NMP22 (89.8%) when applying this interpretation strategy. When considering combinations positive only in case of all markers positive, specificities were increased compared to the single markers with decreased sensitivities. This strategy also led to an increase of accuracy compared to the single tests. The highest specificity and accuracy were observed for the combination of cytology and uCyt+ (specificity 86.2%, accuracy 77.0%).

### 3.3. Combination of Three and Four Tests

Sensitivities, specificities, NPVs, and PPVs for combinations of three and four markers are shown in Table 4. In accordance with the observations made for the two-marker combinations, the addition of a third or a fourth marker increased sensitivity when considering the combination positive if one marker was positive (with decreased specificity). When demanding all markers in the combination to be positive, specificity and accuracy increased but sensitivity dropped markedly.

### 3.4. Stepwise Testing

As the performance of multiple markers is associated with a dramatic increase in costs, we also investigated the performance of a stepwise testing. We performed stepwise testing for the two-marker combinations with highest accuracy (when considering a combination positive if one marker or more were positive), which were cytology and uCyt+ and cytology and FISH. For stepwise testing, the marker associated with lower costs (cytology) was determined first. Results of stepwise testing are shown in Table 4(b). In summary, the performance of FISH and uCyt+ in cytology-negative patients led to the detection of 19 and 14 additional tumors compared to cytology alone. Of those, three (uCyt+) and four (FISH) were high-risk tumors (≥pT1 or G3 or Cis).

## 4. Discussion

The follow-up intervals for cystoscopy after endoscopic resection of a bladder tumor are still discussed controversially [9, 17]. Although cystoscopy has undergone several technical modifications to improve both image quality and tolerability, it is still a relatively painful procedure for many patients [4, 18]. This affects the compliance of the patients and strengthens the need for noninvasive measurements for early detection of bladder cancer recurrence [4]. Noninvasive methods include the application of ultrasound and urine markers with the aim to extend cystoscopy intervals [19].

Due to conflicting data and a low proportion of prospectively designed trials, urine markers are not recommended to replace cystoscopy in current guidelines [2, 8]. The only marker, which is recommended as an adjunct to cystoscopy in current guidelines, is cytology, showing acceptable sensitivity only for high-grade tumors [2]. To date, only little is known on the performance of combined use of cell- and protein-based urine tests to detect bladder cancer recurrence [9]. The aim of the present study was to assess the performance of four broadly available markers and their combinations in a large cohort of patients undergoing surveillance for bladder cancer. As combining urine markers leads to a significant increase in costs, we also aimed to investigate the performance of these tests when applied in a stepwise manner.

Our results show that the way a combination of urine marker changes the sensitivity and specificity compared to the single tests mainly depends on the interpretation strategy. When considering a combination positive if only one of the tests included is positive, a clear improvement of sensitivity can be observed. However, this is associated with a clear decrease in specificity and accuracy. When considering a combination positive only in the case of all markers being positive, this leads to an improvement of specificity and accuracy, but sensitivity drops clearly. These results raise the question, whether sensitivity or specificity is more important for a urine marker in the follow-up of patients with NMIBC. Although specificity is important to prevent false positive tests, we consider a high sensitivity and a high negative predictive value to be more important in the context of follow-up for NMIBC. Although a marker or combination with high specificity would spare a higher proportion of patients from cystoscopy, the most important feature, also for counseling the patient, should be a high detection rate particularly for high-risk tumors. A high negative predictive value, meaning a low risk of having a tumor when the test or combination is negative, is the main prerequisite for preventing missed tumors potentially progressing to a muscle invasive stage. A marker showing a specificity of 50% but a high NPV has the potential to supersede a planned cystoscopy in 50% of patients without evidence of tumor (when taking into account before performing follow-up cystoscopy).

In the present study, we observed that combinations of two markers, applied either simultaneously or stepwise, can achieve considerably high sensitivities especially for high-risk tumors. In the case of a stepwise application of cytology and FISH, the sensitivity was 80.5% with sensitivity for high-risk tumors of 94%. This is in accordance with other studies showing the UroVysion test to be a good adjunct to cytology in the detection of high-grade tumors. Gudjonsson et al. investigated the role of UroVysion in the surveillance of NMIBC. In their study, two of five cases with carcinoma in situ were only detected by FISH with no clear evidence of malignancy in cytology [20]. An even more dramatic increase in the detection rate of high-risk tumors when performing FISH in addition to cytology has been observed in a study by Galvan et al. In their study including 223 patients undergoing surveillance of NMIBC 10/14 high-risk recurrences were detected only by the means of FISH. Different results were obtained by a study from Netherlands showing a clearly worse performance of FISH compared to cytology in the detection of recurrences during surveillance of NMIBC. In this study, the combination of both markers could only detect 53.1% of recurrences [21].

An even higher overall sensitivity in our study of 84.4% could be reached by the combination of cytology and immunocytology. The tumors additionally detected by immunocytology were mainly (16/19) low risk tumors. The sensitivity of the combination of cytology and immunocytology for high-risk tumors was 92.0%. These results are similar to a study from Bolzano investigating the performance of cytology and immunocytology in a cohort of 216 patients undergoing surveillance for NMIBC. They observed an overall sensitivity of 86.6% for the combination of cytology and immunocytology. For high-risk tumors, the sensitivity was 89.2% [22]. Another study performed by Sullivan et al. in a cohort of 100 patients showed a sensitivity of the combination of immunocytology and FISH of 75% (91% for G3 tumors and 100% for T1 tumors). Similar to our study, the addition of immunocytology to cytology led to a clear worsening of specificity (62%) compared to cytology alone (97%).

In our study, 7% of tumors and 4% of high-risk tumors were exclusively NMP22 positive. No other markers achieved a higher rate of tumors solely positive for one marker. However, NMP22 had a relatively low specificity in our study both as a single marker and in combination with other markers. This is in contrast to other studies reporting specificities of over 70% for both NMP22 as a single marker and in combination with cytology [5, 23]. However, most of these studies used the NMP22 BladderChek point-of-care test. This might partially account for the differences in the results, as the ELISA and the point-of-care test system use completely different techniques for protein concentrations and are therefore critical to compare.

The performance of multiple markers is associated with relatively high costs (depending on the markers) [24]. Reimbursement data by Medicare 2009 show that the costs for a FISH UroVysion test (including technical and professional components) are almost three times as high as an office cystoscopy ($275) and almost 9 times as high as cytological evaluation ($90 including microscopy). One way to partially reduce the costs associated with multiple marker testing is a stepwise approach. Due to the broadly observed high specificity of cytology and its relatively low costs, combinations with cytology in the first step should be sought.

Combinations of three and four markers led to a further increase of sensitivity in our study. Sensitivities for overall tumor detection and high-risk tumor detection reached considerable 94.5 and 100% when combining FISH and uCyt+ and NMP22. However, only one of the four possible three-marker combinations (cytology, FISH, and uCyt+) achieved a specificity >50% (when considering a combination positive if at least one marker is positive).

There are some limitations to be stated. The main limitation of our study is that no follow-up information is available for the included patients. Therefore, the effect of anticipatory-positive urine markers could not be addressed [25]. Moreover, the study population was relatively heterogonous with regard to interval between urine sampling and last evidence of tumor. This might have also affected the results in the study. For a high proportion of patients, grading was performed based on the WHO classification from 1973. Another important limitation is that we did not control for the type of tumor found in last transurethral resection of a bladder tumor (TUR-BT), which might have also affected the present results. In contrast to other studies, sensitivity of cytology is far higher which might be the result of an experienced investigator for urine cytology.

What are the clinical implications of the results of our study? The present results show that expedient combinations of urine markers have the potential to detect a high percentage of bladder cancer recurrences and harbor a very low risk of missing a high-risk tumor. This might form the basis of using them in selected patients with pronounced stress and fear caused by routine follow-up cystoscopy. Although this issue was not specifically addressed in our study, we do not expect this approach to be more cost-effective than a cystoscopy-based follow-up due to the relatively high costs especially for the UroVysion FISH assay. Prior studies have shown that cystoscopy alone remains the most cost-effective strategy to detect recurrences of bladder cancer [26]. However, the decision to use urine markers in the follow-up of patients with NMIBC should only be based on cost-effectiveness but should always account for the degree of stress and pain an individual patient has from cystoscopy.

## 5. Conclusions

This is the first study assessing the use of four broadly used urine markers in the surveillance of patients with NMIBC. Performing a simultaneous or stepwise application of urine markers can lead to a considerable improvement of sensitivity. The risk of missing a tumor, particularly a high-risk tumor (G3, Cis, pT1, and higher), is very low when performing two-test combinations of cytology, uCyt+, and FISH. The high sensitivity of these combinations justifies their use in prospective trials assessing the use of urine markers to individualize the cystoscopy follow-up in patients with NMIBC.

## Figures and Tables

**Table 1:** Patients' characteristics.

Total number of patients	483
Median age (range)	70 (31–94)
Gender (male/female)	406/77
Tumor at time of urine marker sampling *n* (%)	128 (26.5)
pTa *n* (%)	86 (67.2)
pT1 *n* (%)	15 (11.7)
≥pT2 *n* (%)	14 (10.9)
Pure Cis *n* (%)	13 (10.1)
Concomitant Cis *n* (%)	8 (6.3)
G1 *n* (%)	53 (42.4)
G2 *n* (%)	38 (29.7)
G3 *n* (%)	25 (19.5)
Interval between last tumor and urine marker sampling (median, range; months)	6 (2–94)

**Table 2:** Single test performances in detecting recurrences and high risk recurrences in patients undergoing surveillance of non-muscle-invasive bladder cancer (NMIBC). Values are given in %. Pos: positive, PPV: positive predictive value, NPV: negative predictive value, FISH: fluorescence in situ hybridization, and NMP22: nuclear matrix protein 22.

Test	*n* pos	Sensitivity total (sensitivity high risk tumors)	Specificity	PPV	NPV total (NPV high risk tumors)	Accuracy
Cytology	176	69.5 (86.0)	75.5	50.5	87.3 (97.7)	73.3

FISH	203	74.3 (90.0)	69.6	46.8	88.2 (98.2)	70.8

uCyt+	191	66.4 (72.0)	70.1	44.5	85.3 (95.2)	69.2

NMP22	268	70.3 (80.0)	49.9	33.6	82.3 (95.3)	55.4

**Table 3:** Performance of marker combinations in detecting recurrences in patients undergoing surveillance of non-muscle-invasive bladder cancer (NMIBC). Values are given in %. Two ways of considering marker combinations as positive were applied. In the first analysis, every positive marker led to a positive combination. In the second analysis, all markers had to be positive for considering a combination positive. High-risk tumors were defined as tumors showing at least one of the following features: pT1 or higher, G3, carcinoma in situ (Cis). Pos: positive, Sens: sensitivity, Spec: specificity, PPV: positive predictive value, NPV: negative predictive value, FISH: fluorescence in situ hybridization, and NMP22: nuclear matrix protein 22.

Combination	Criteria for positive combination	*n* pos	Sensitivity total (sensitivity high risk tumors)	Spec	PPV	NPV total (NPV high risk tumors)	Accuracy
2-Test combinations							
Cytology & FISH	≥1 marker pos	230	80.5 (94.0)	64.2	44.7	90.1 (98.8)	68.5
	2 markers pos	149	63.3 (82.0)	80.8	54.3	85.9 (89.6)	76.2
Cytology & uCyt	≥1 marker pos	252	84.4 (92.0)	59.5	42.8	91.3 (98.2)	66.1
	2 markers pos	115	51.6 (66.0)	86.2	57.4	82.3 (95.3)	77.0
Cytology & NMP22	≥1 marker pos	318	86.7 (98.0)	40.8	34.6	89.5 (97.7)	53.0
	2 markers pos	123	53.1 (68.0)	84.5	55.3	83.3 (95.0)	76.2
FISH & uCyt	≥1 marker pos	269	86.7 (96.0)	56.5	41.5	92.1 (98.2)	63.7
	2 markers pos	125	53.9 (66.0)	84.3	55.2	83.5 (95.2)	76.2
FISH & NMP22	≥1 marker pos	330	88.3 (96.0)	39.2	33.2	90.2 (98.6)	51.9
	2 markers pos	141	56.3 (74.0)	80.6	51.1	83.6 (96.1)	74.1
uCyt & NMP22	≥1 marker pos	335	89.8 (92.0)	38.1	34.3	91.2 (97.2)	51.8
	2 markers pos	124	46.8 (60.0)	82.0	48.4	81.1 (94.4)	72.7
3-Test combinations							
Cytology & FISH & uCyt+	≥1 marker pos	282	87.5 (96.0)	52.1	39.7	92.0 (99.0)	61.4
	3 markers pos	101	46.1 (62.0)	88.2	58.4	81.9 (95.0)	77.0
Cytology & FISH & NMP22	≥1 marker pos	338	90.6 (98.0)	37.5	34.3	91.7 (99.3)	51.5
	3 markers pos	104	49.2 (66.0)	88.5	60.0	82.8 (95.5)	78.1
Cytology & uCyt & NMP22	≥1 marker pos	352	93.8 (100.0)	34.7	34.1	93.8 (100.0)	50.3
	3 markers pos	79	39.1 (56.0)	91.2	63.3	80.7 (94.5)	77.8
FISH & uCyt & NMP22	≥1 marker pos	356	94.5 (100.0)	33.8	36.0	94.5 (100.0)	49.9
	3 markers pos	84	40.6 (58.0)	91.0	61.9	80.9 (94.7)	77.6
4-Test combination							
Cytology & FISH & uCyt+ & NMP22	≥1 marker pos	359	94.5 (100.0)	33.1	33.7	94.4 (100.0)	49.3
	4 markers pos	68	35.9 (54.0)	93.3	65.7	80.1 (94.4)	78.1

**(a):** 

Combination positive (*n*)/negative (*n*)/specificity (%)	230/253/64.2%
Tumors detected (*n*)/sensitivity (%)	103/80.5%
High-risk tumors detected (*n*)/sensitivity (%)	47/94%
Numbers of tumors missed (*n*)/NPV (%)	25/90.1%
Numbers of high-risk tumors missed (*n*)/NPV (%)	3/98.8%
Numbers of false positive tests (*n*)/PPV (%)	127/44.7%
Additional tumors detected (*n*)/additional high-risk tumors detected by uCyt+ (*n*)	14/4

**(b):** 

Combination positive (*n*)/negative (*n*)/specificity (%)	231/252/59.5%
Tumors detected (*n*)/sensitivity (%)	108/84.4%
High-risk tumors detected (*n*)/sensitivity (%)	46/92.0%
Numbers of tumors missed (*n*)/NPV (%)	20/91.3%
Numbers of high-risk tumors missed (*n*)/NPV (%)	4/98.8%
Numbers of false positive tests (*n*)/PPV (%)	144/42.8%
Additional tumors detected (*n*)/additional high-risk tumors detected (*n*)	19/3
